# Hepatic bioenergetics and metabolism in mitochondrial disease: insights from the *Ndufs4* KO mouse model

**DOI:** 10.1007/s11306-025-02275-7

**Published:** 2025-06-11

**Authors:** Karin Terburgh, Nastassja Sweeney, Roan Louw

**Affiliations:** Human Metabolomics, Faculty of Natural and Agricultural Sciences, North-West University, (Potchefstroom Campus), Private Bag, Potchefstroom, X6001 South Africa

**Keywords:** *Ndufs4* knockout mice, Leigh syndrome, Complex I deficiency, Liver metabolism

## Abstract

**Introduction:**

Mitochondrial complex (CI) deficiency frequently manifests as a severe neurometabolic disorder called Leigh syndrome (LS). Research on the *Ndufs4* knockout (KO) mouse model has identified neuronal vulnerability to CI deficiency as a major driver of the disease, yet its effects on hepatic function remain unclear. Considering the importance of the liver, and its interconnection with the brain, in regulating whole-body metabolic balance, further investigation into the effects of whole-body *Ndufs4* KO on the liver is warranted.

**Objectives:**

This study investigated liver bioenergetics and metabolism in *Ndufs4* KO and WT mice at the late stage of LS.

**Methods:**

Bioenergetic investigations of liver mitochondria (*n* ≥ 3) included spectrophotometric respiratory chain enzyme (CI-IV) activity assays and high-resolution respirometry. Hypothesis-generating metabolomics of whole-liver extracts (*n* ≥ 19) utilised ^1^H-NMR, GC-TOFMS, and LC-MS/MS. Significant alterations were identified via t-tests and effect size calculations.

**Results:**

*Ndufs4* KO livers displayed a significant ~ 86% reduction in CI activity and a ~ 43% decrease in CI contribution to CI + II-driven respiration. CII-driven respiration remained unaffected, providing the predominant electron flux in both genotypes. Metabolic profiling revealed widespread perturbations in *Ndufs4* KO hepatic metabolism including glucose-, amino acid-, purine/pyrimidine metabolism and the TCA-cycle.

**Conclusion:**

Despite severe CI deficiency, respiration in the *Ndufs4* KO liver remains largely unaffected due to reliance on CII. Nonetheless, advanced LS significantly disrupts liver metabolism, with O-GlcNAcylation and mTOR signalling suggestsed as key areas for future investigation. Altogether, our findings underscore the importance of interorgan metabolic dynamics and the liver-brain axis in neurometabolic disorders like LS.

## Introduction

Mitochondrial disorders exhibit a broad spectrum of clinical manifestations, affecting either single or multiple organ systems. The liver, a central hub of metabolism, plays a pivotal role in maintaining whole-body homeostasis and extensively interacts with multiple organs — contributing to digestion, metabolism, detoxification, vitamin and mineral storage, and immune responses. Liver involvement is common in mitochondrial diseases, with patients frequently presenting with hepatomegaly, steatosis, cholestasis, and liver failure (Rahman, 2013). However, isolated complex I (CI) deficiency, one of the most prevalent forms of mitochondrial disease, does not typically result in overt liver phenotypes (Diaz et al., 2011; Distelmaier et al., 2009; Lesner et al., 2022).

In paediatric patients, CI deficiency most commonly presents as Leigh syndrome (LS) — a progressive neurometabolic disorder characterised by bilaterally symmetric brain lesions (Lake et al., 2016; Loeffen et al., 2000). The *Ndufs4* knockout (KO) mouse model of LS (JAX, 2017; Kruse et al., 2008) has become an essential tool for investigating the tissue-specific consequences of mitochondrial CI deficiency with most research focused on the brain and the vulnerability of neurons to CI deficiency (van de Wal et al., 2022). The effects of CI deficiency on the liver, however, is less clear.

Despite the critical role of CI in cellular respiration, its function in the liver seems to differ from that in other tissues. Some studies suggest that CI is dispensable for liver function, as CI-deficient mouse livers do not show signs of dysfunction or altered bioenergetics (Balmaceda et al., 2024; Lesner et al., 2022). Conversely, evidence from *Ndufs4* KO mice indicates that the liver plays a crucial role in mediating some of the systemic effects of CI-related pathologies. Liver-specific CI deficiency results in increased serum triglycerides, lactate, and bone mass (Jin et al., 2014); while whole-body CI deficiency leads to increased liver inflammatory markers (Jin et al., 2014), a deficiency in liver lipids and free fatty acids (Johnson et al., 2013), decreased liver glutamine anaplerosis and circulatory turnover flux (Yang et al., 2020), and liver iron overload (Kelly et al., 2023). Notably, treatments targeting the liver have shown neurological benefits in *Ndufs4* KO mice. For instance, the liver-specific disruption of S6K1, a key effector in the mTOR pathway, enhanced neuronal survival and moderately extended lifespan in these mice — benefits not observed with neuron- or adipocyte-specific S6K1 disruption (Ito et al., 2017). Additionally, dietary iron restriction reduced neuroinflammation and improved survival in *Ndufs4* KO mice (Kelly et al., 2023).

These findings raise intriguing questions about the liver’s capacity to maintain metabolic homeostasis and compensate for the loss of CI function as LS progresses. Given the liver’s central role in systemic health and its interplay with the brain in regulating whole-body metabolic balance, understanding the impact of whole-body CI deficiency on hepatic bioenergetics and metabolism is critical. Here, we utilise enzyme assays, high-resolution respirometry, and hypothesis-generating multiplatform metabolomics to explore the interplay between hepatic bioenergetics, metabolism, and whole-body CI dysfunction in the *Ndufs4* KO mouse model at the late stage of LS.

## Methods

### Animals and sampling

*Ndufs4* KO and age-matched WT mice originating from B6.129S4-*Ndufs4*^*tm1.1Rpa*^/J (JAX #027058) heterozygous crosses, were bred and housed at the NWU Preclinical Drug Development Platform (SAVC reg. #FR15/13458). All protocols were approved by NWU Animal Care, Health and Safety Research Ethics Committee (#NWU-00430-21-A5, # NWU-00001-15-A5, and #NWU-0509-20-A1). Mice were housed under standard conditions (12:12 h light/dark cycle, 22 ± 1 °C, 55 ± 10% humidity) with *ad libitum* access to Rodent Breeder chow (#RM1845) and water. Genotypes were confirmed by PCR from tail snips, with only male animals used to control for hormonal and metabolic variability (Ruoppolo et al., 2018; Wells et al., 2016). Mice were euthanised by cervical dislocation at postnatal day (P) 45–50, when mice exhibited severe disease symptoms (Quintana et al., 2010). This age range reflects late-stage LS, as determined by previous survival and phenotypic data from our facility (Miller et al., 2021). Freshly collected tissues were immediately processed for bioenergetic analysis (*n* ≥ 3 biological repeats per group). For metabolic analyses (*n* ≥ 19 biological repeats per group), tissues were harvested at the same time of day following a 12-hour fast. Immediately post-euthanasia, liver samples were snap-frozen in liquid nitrogen within seconds and stored at -80 °C as previously described (Terburgh et al., 2021).

### Mitochondrial isolation

Liver mitochondria were isolated using differential centrifugation, following the protocol of Sumbalova et al. (2016) with minor modifications. Fresh liver tissue (~ 1.5 g) was dissected, minced, and homogenised in ice-cold isolation buffer (225 mM mannitol, 75 mM sucrose, 0.2 mM EDTA, pH 7.4) using a Dounce grinder with a loose fit pestle (8–10 strokes, moderate speed). Thereafter, the homogenate was centrifuged at 1000 xg for 10 min at 4 °C, followed by centrifugation of the supernatant at 6200 xg for 10 min at 4 °C. The mitochondrial pellet was then resuspended in isolation buffer (8 mL/g tissue), centrifuged at 6200 xg for 10 min at 4 °C, with the pellet finally resuspended in isolation buffer (1 mL/g tissue). A portion of the mitochondria was used immediately for respirometry, while the remainder was stored at -20 °C for subsequent enzyme assays.

### Respiratory chain enzyme assays

Kinetic spectrophotometric assays for complexes I-IV of the respiratory chain and citrate synthase (CS) were performed on a Synergy™ HT reader (Biotek Instruments) according to established protocols (Janssen et al., 2007; Luo et al., 2008; Rahman et al., 1996; Shepherd & Garland, 1969). Mitochondrial isolates underwent three freeze-thaw cycles to disrupt mitochondrial membranes after which assays were conducted in triplicate at 37 °C using 96-well UV microplates. Data were analysed using Gen5™ Data Analysis software (v 1.11.5), with enzyme activities normalised to units of citrate synthase (UCS in µmol/min/mg protein) to account for mitochondrial content.

### Respirometry

Respiration was measured in isolated liver mitochondria using the Oroboros O2k high-resolution respirometer (Oroboros Instruments) following the SUIT-008 O2 mt D026 protocol. After air calibration, measurements were performed at 37 °C in MiR05 buffer. Substrates and inhibitors were added sequentially to assess isolated and combined complex I-driven (pyruvate, malate, ADP, glutamate) and complex II-driven (succinate, rotenone) respiration. Data were analysed using DatLab software. Raw oxygen consumption rates were normalised to units of citrate synthase (UCS in µmol/min/mg protein) to account for mitochondrial content, while oxygen flux values were corrected for instrumental background and residual oxygen consumption. CI and CII control ratios were respectively calculated by dividing the respiration rates of each complex when active in isolation by the total respiration rate measured when active simultaneously. These ratios were expressed as CI/(CI + II) and CII/(CI + II), respectively.

### Metabolic profiling

Metabolic profiling was conducted with minor modifications to established methods (Terburgh et al., 2019) as detailed below.

####  Metabolite extraction

Liver tissues were briefly thawed on ice and finely sliced into ~ 65–75 mg sections using sterile scissors on a pre-chilled (4 °C) metal surface. Samples were extracted using a single-phase Bligh-Dyer method (Gullberg et al., 2004) with ice-cold methanol/water/chloroform (3:1:1, v/v/v). Homogenisation was performed using two 3 mm beads per sample in a vibration mill at 20 Hz for 2 min. The aqueous phase used for extraction included internal standards (1 µg/mg tissue): *N*,*N*-dimethyl-L-phenylalanine, 3-phenylbutyric acid, and norleucine. Extracts were divided for analysis via ¹H-NMR (250 µL), GC-TOFMS (100 µL), and LC-MS/MS (100 µL) with quality control (QC) samples generated by pooling sample aliquots. An isotopically labelled internal standard mixture was added to each LC sample prior to drying all samples under a stream of nitrogen gas and storage at -80 °C.

#### ¹H-NMR analysis

Untargeted ¹H-NMR was performed using a 500 MHz Bruker Avance III HD NMR spectrometer equipped with a 5 mm TXI probe and gradient coils. Samples were prepared in 2 mm NMR tubes using an eVol digital syringe. Dried extracts were resuspended in 60 µL HPC-grade H_2_O, centrifuged (12 000 xg), and the supernatant mixed with NMR buffer (K H_2_PO_4_, TSP-d4, NaN_3_ in D_2_O, pH 7.4) in a 10:90 D_2_O: H_2_O ratio. Spectra were acquired at 300 K using a NOESY-presat pulse sequence (4 s relaxation delay, 10 µs 90° pulse) with H_2_O suppression at 4.7 ppm. Parameters included 128 scans, 32 K data points, 12 ppm spectral width, 2.7 s acquisition time, and 64 receiver gain. Automated shimming, locking, and calibration were performed prior to each acquisition. Spectra were processed in Bruker Topspin (v3.5) and analysed in AMIX (v3.9.14). Metabolites were identified by comparison with spectral libraries, with additional confirmation using 2D ¹H-¹H correlation and J-resolved spectroscopy.

####  GC-TOFMS analysis

Untargeted GC-TOFMS was performed using an Agilent 7890 A GC coupled to a LECO Pegasus HT TOF mass spectrometer. Dried extracts underwent sequential derivatisation with 50 µL 20 mg/mL methoxyamine hydrochloride in pyridine (60 °C, 60 min) followed by 50 µL BSTFA + 1% TMCS (60 °C, 60 min). Samples (1 µL) were injected in split mode (1:5) onto an Rxi-5Sil MS capillary column (28.6 m × 250 μm × 0.25 μm). The temperature program initiated at 70 °C (1 min hold), followed by gradients of 7 °C/min to 120 °C, 10 °C/min to 230 °C, and 13 °C/min to 300 °C (final 1 min hold). The transfer line and ion source temperatures were maintained at 225 °C and 200 °C, respectively. Mass spectrometry was performed in electron ionization mode (-70 eV) with a 250 s solvent delay, acquiring data at 20 spectra/s across m/z 50–950. Data were processed using LECO ChromaTOF (v4.5x), with peak alignment performed via the Statistical Compare module. Metabolite identification was achieved by matching mass spectra and retention times against commercial and in-house libraries, applying a minimum spectral similarity threshold of 80%.

####  LC-MS/MS analysis

Semi-targeted LC-MS/MS analysis of acylcarnitines, amino acids and their derivatives was performed using an Agilent 1260 LC system coupled to a 6470 triple quadrupole mass spectrometer. Dried extracts were derivatised with butanolic-HCl (1-butanol: acetyl chloride, 4:1 v/v) at 50 °C for 60 min, evaporated to dryness, and reconstituted in 100 µL of 50:50 (v/v) water: acetonitrile containing 0.1% formic acid. Chromatographic separation was achieved on a Zorbax SB-Aq column (100 × 2.1 mm, 1.8 μm) maintained at 45 °C using a binary gradient of 0.1% formic acid in (A) water and (B) acetonitrile at 0.3–0.4 mL/min: initial 95% A (0.2 min), ramp to 25% B (1.8 min), hold (5 min), increase to 90% B (0.5 min), hold (1.6 min), ramp to 95% B (2.9 min), then re-equilibrate (4 min). Positive electrospray ionisation was performed with the following parameters: 300 °C source temperature, 7.5 L/min drying gas flow, 30 psi nebuliser pressure, and 3500 V capillary voltage. Data were acquired using MassHunter B02.01 and processed with B06.00. Metabolites were identified by monitoring two unique multiple reaction monitoring (MRM) transitions per analyte with spectral and retention time confirmation against reference standards analysed concurrently.

####  Data processing

Data from each platform underwent automated baseline correction and peak alignment using the appropriate software, followed by manual inspection to ensure accurate peak picking and quality. The data were then preprocessed in Excel to remove unreliable features with high missing values (modified 80% rule); high QC-CV values (> 25% for targeted data and > 50% for untargeted data) and high variance (Fisher ratio < 0.05 (Pierce et al., 2006). Features were normalised to internal standards, which were added according to tissue mass, with the data subsequently log transformed and auto scaled.

### Data analysis

Statistical analyses were performed using two-tailed t-tests, with p-values adjusted for multiple testing where applicable, using Bonferroni-Holm false discovery rate (FDR) corrections in MetaboAnalyst (Xia Lab, version 5.0) (Pang et al., 2021; Xia et al., 2009). Effect sizes (Cohen’s d-value) were calculated in Excel (Microsoft 365) as an indicator of practical significance. For untargeted analyses, only metabolites exhibiting significant alterations were structurally confirmed. Confidence levels in metabolite identification followed the Schymanski et al. (2014) framework, with assignments supported by spectral matching, retention time, and/or reference standards as applicable. Boxplots were generated for bioenergetic data using BoxPlotR (Spitzer et al., 2014), displaying the minimum and maximum values, inter-quartile range, and median for each group. Multivariate analyses, including principal component analysis (PCA) and hierarchical clustering, conducted in MetaboAnalyst on combined datasets, with duplicate metabolites removed.

## Results

### Respiratory chain enzyme activity and respirometry

**Fig. 1:**
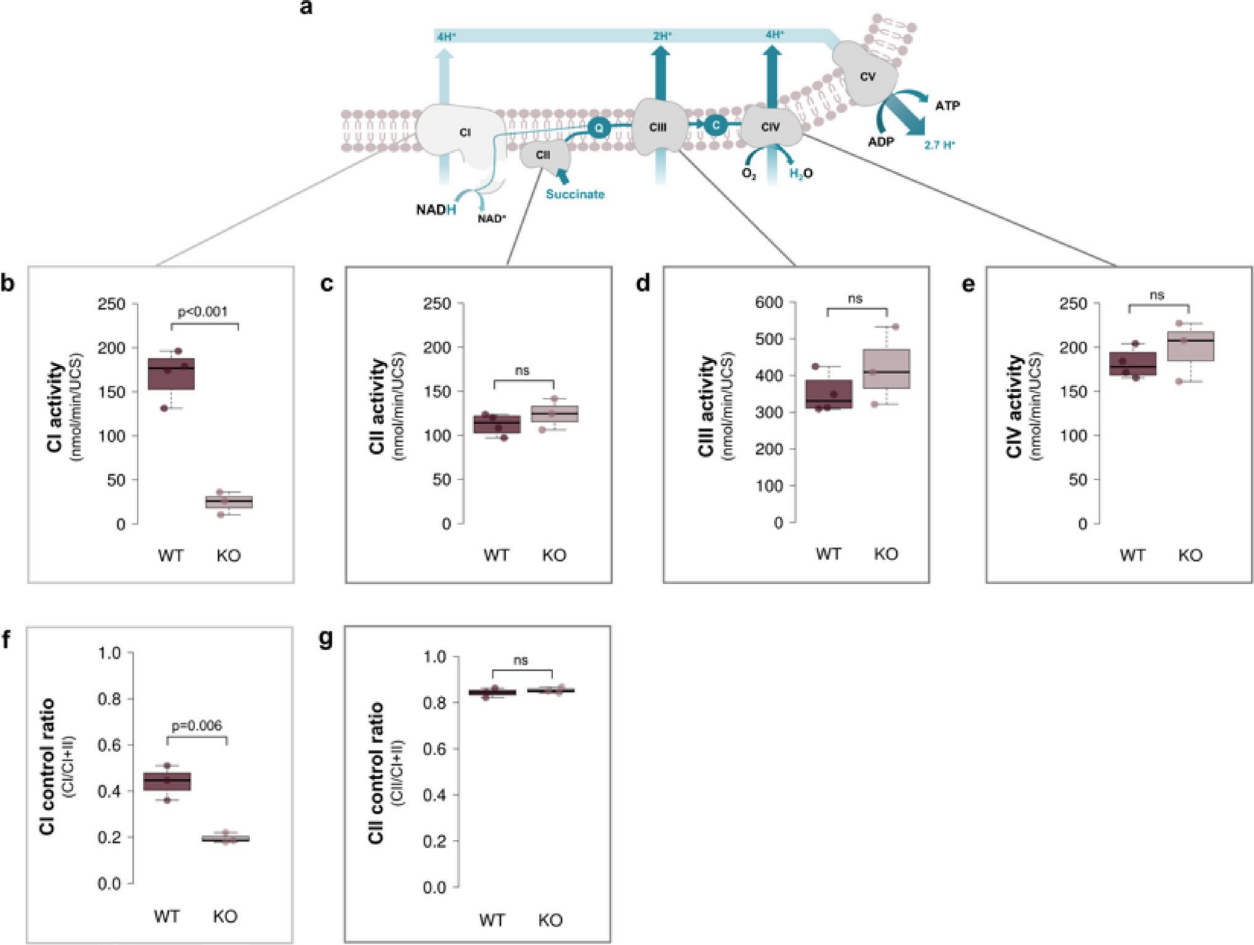
Respiratory chain enzyme activity and respiratory control ratios in the liver of *Ndufs4* KO vs. WT mice. **a** Schematic representation of the mitochondrial OXPHOS system. **b**–**e** Maximal enzyme activities of complexes I-IV in mouse liver mitochondria, normalized to units of citrate synthase (UCS). Each data point represents the average of triplicate measurements from WT (*n* = 4) and KO (*n* = 3) mice. **f**–**g** Control ratios for CI and CII in liver mitochondria from WT (*n* = 3) and KO (*n* = 3) mice. CI control ratios were determined by dividing the oxygen consumption rates from isolated CI-driven respiration (after the addition of pyruvate, malate, ADP, and glutamate) by those from convergent CI + II-driven respiration (after the addition of pyruvate, malate, ADP, glutamate, and succinate). CII control ratios were determined by dividing the oxygen consumption rates from isolated CII-driven respiration (after the addition of pyruvate, malate, ADP, glutamate, succinate, uncoupler, and rotenone) by the oxygen consumption rates from convergent CI + CII-driven respiration (after the addition of pyruvate, malate, ADP, glutamate, succinate, and uncoupler). The significance of genotypic differences was evaluated using a two-way Student’s t-test. Box plots display minimum and maximum values, the inter-quartile range (box height), and median (thick horizontal line) for each group. *CI* complex I, *CII* complex II, *CIII* complex III, complex IV; *KO* knockout; *UCS* units of citrate synthase; *WT* wildtype

To assess the effect of the *Ndufs4* KO on the respiratory chain, the maximal catalytic activity of respiratory chain enzymes, CI-CIV of the OXPHOS system (Fig. 1a), along with CI- and CII-driven respiration were assessed in liver mitochondria from WT (*n* ≥ 3) and *Ndufs4* KO (*n* = 3) mice. Spectrophotometric enzyme assays confirmed an 86% decrease (*p* = 0.0004, D = 5.27) in rotenone-sensitive CI activity (Fig. 1b) in *Ndufs4* KO compared to WT livers. In contrast, CII-CIV activities (Fig. 1c-e) were unaffected by the *Ndufs4* KO. High resolution respirometry further revealed a marked decrease in the contribution of CI to combined CI + II-driven respiration in the *Ndufs4* KO liver (Fig. 1f), with the CI/CI + II control ratio declined from 0.44 in WT livers to 0.19 in *Ndufs4* KO livers (*p* = 0.006, D = 3.25). Thereagainst, the CII control ratio (Fig. 1g) was unaltered, remaining at ~ 0.85 and contributing to the majority of the overall electron flux in both WT and *Ndufs4* KO liver mitochondria.

### Metabolic profiling

**Fig. 2:**
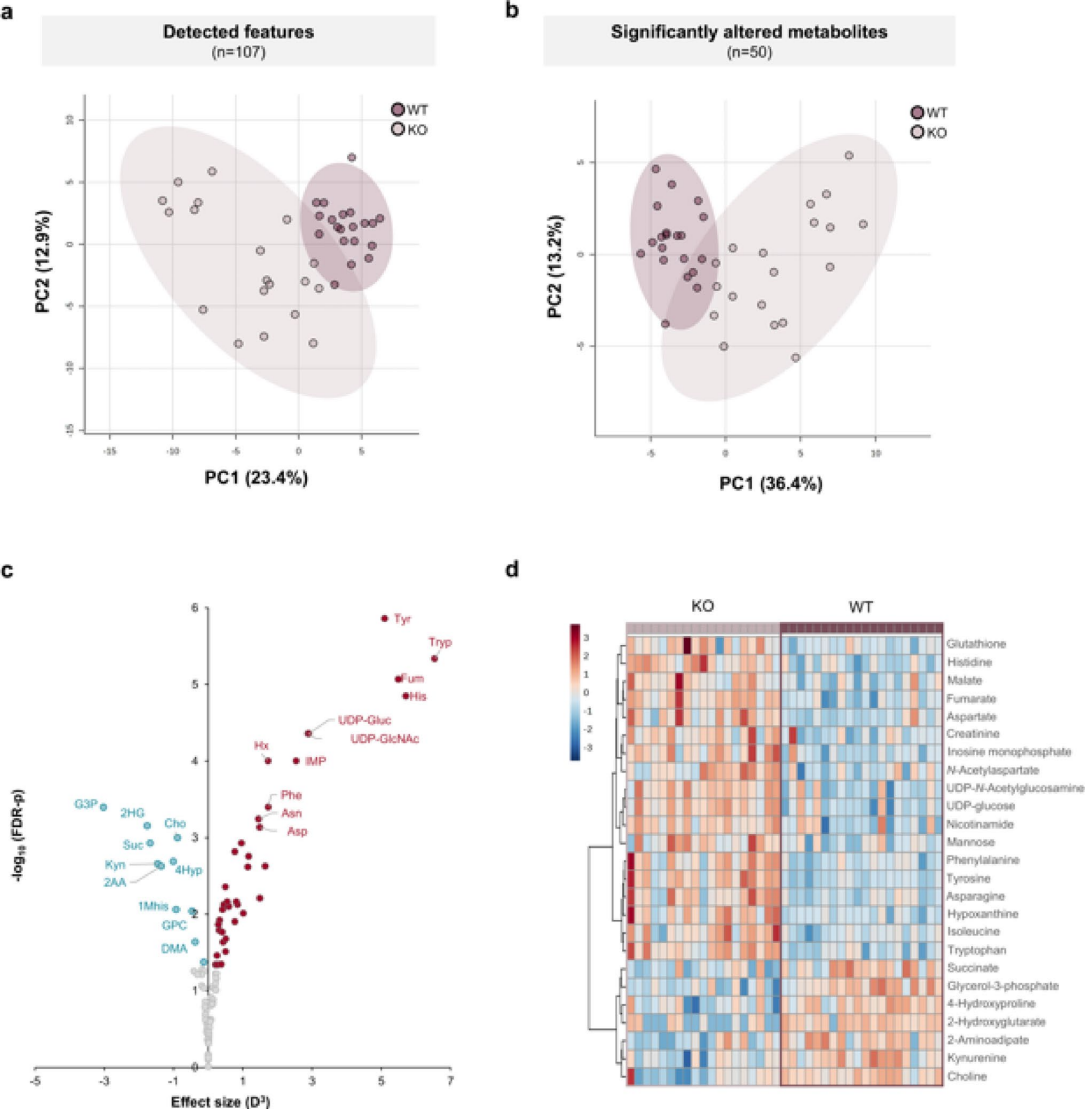
Metabolic profiling of *Ndufs4* KO vs. WT mouse livers. **a**–**b** Principal component analysis (PCA) of metabolites detected in WT and KO mouse livers using LC-MS/MS, GC-TOFMS, and ^1^H-NMR spectroscopy shown before (a) and after (**b**) feature selection. The first two principal components are plotted, with ellipses representing 95% confidence intervals for each group. Data were log-transformed and auto scaled (*n* ≥ 19 per group). **c** Volcano plots displaying the relationship between statistical significance (-log_10_ FDR-corrected p-values) and effect size (cubed Cohen’s D) for metabolites differentiating KO from WT livers. Metabolites significantly reduced or elevated in KO compared to WT are represented by blue and red filled circles, respectively (FDR-corrected *p* < 0.05 and D ≥ 0.5). Non-significant features are shown in grey. For untargeted GC data, unknown features were excluded. **d** Hierarchical clustering heatmap illustrating the relative abundance of metabolites across samples. Clustering was conducted using the Euclidean distance metric and Ward’s linkage method, focusing on the 24 metabolites with the lowest t-test p-values. For untargeted GC data, unknown features were excluded. *1Mhis* 1-methylhistidine; *2AA* 2-aminoadipate, *2HG* 2-hydroxyglutarate, *4Hyp* 4-hydroxyproline, *Asn* asparagine, *Asp* aspartate, *Cho* choline, *DMA* dimethylamine, *Fum* fumarate, *G3P* gycerol-3-phosphate, *GPC* glycerophosphocholine, *His* histidine; *Hx* hypoxanthine,* IMP* inosine monophosphate, *Kyn* kynurenine, *Phe* phenylalanine, *Suc* succinate, *Tryp* tryptophan, *Tyr* tyrosine; *UDP-GlcNAc* UDP-N-acetylglucosamine, *UDP-Gluc* UDP-glucose

To gain insight into the areas of liver metabolism affected by the *Ndufs4* KO, multi-platform metabolic profiling was employed as a hypothesis-generating tool. After data filtering and removal of duplicate compounds from combined datasets, a total of 107 features were detected in the livers of 45 to 50-day-old mice, with 50 metabolites significantly affected by the *Ndufs4* KO (Table 1). Multivariate principal component analysis (PCA) of the metabolic data revealed natural separation between genotypes, though some overlap in the scores was observed. The metabolic differences attributable to genotype were predominantly captured by the first principal component (PC1), which accounted for 23.4% of the total variance when all detected features were considered (Fig. 2a). This variance increased to 36% when the analysis was restricted to significantly altered metabolites (Fig. 2b). A volcano plot (Fig. 2c) was generated to visualise the statistical significance [-log_10_(FDR-p)] relative to the magnitude of change (effect size cubed) in metabolite levels between genotypes. This plot revealed a significant increase in the levels of most metabolites in *Ndufs4* KO livers compared to WTs (highlighted by red data points), while a distinct cluster of 11 metabolites exhibited a marked decrease (indicated by blue data points). A hierarchical clustering heatmap further displays the differences in abundance of the 24 metabolites with the lowest p-value across samples (Fig. 2d).

**Table 1:** Significantly altered metabolites discriminating between the *Ndufs4* KO and WT mouse liver

Metabolite	Platform	ID level ^a^	t-test *p*	FDR-*p*	Effect size	Direction in KO ^b^	Platform confirmed ^c^
1-Methylhistidine	LC	1	3.49E–03	8.66E–03	0.97	↓	NMR
2-Aminoadipic acid	LC	1	6.52E-04	2.35E–03	1.11	↓	
2-Hydroxyglutarate	GC	2	3.32E-05	6.97E–04	1.21	↓	
β-Alanine	LC	1	1.28E-02	2.31E-02	0.76	↑	
4-Hydroxyproline	LC	1	3.97E-04	2.04E-03	1.00	↓	
Glucose	NMR	1	3.63E-02	4.20E-02	0.50	↓	
Alanine	GC	2	1.02E-02	3.07E-02	0.79	↑	
Asparagine	LC	1	7.92E-05	5.70E-04	1.13	↑	
Aspartic acid	LC	1	1.22E-04	7.29E-04	1.14	↑	GC
Choline	NMR	1	3.17E-04	9.96E-04	0.96	↓	
Citrulline	LC	1	8.06E-03	1.61E-02	0.68	↑	
Creatine	LC	1	5.89E-03	1.25E-02	0.92	↑	
Creatinine	GC	2	1.84E-03	9.70E-03	1.00	↑	
Decanoylcarnitine (C10)	LC	1	9.03E-03	1.71E-02	0.74	↑	
Dimethylamine	NMR	1	1.88E-02	2.30E-02	0.72	↓	
Fumaric acid	NMR	1	7.77E-07	8.54E-06	1.76	↑	GC
Glutathione	NMR	2	6.91E-04	1.52E-03	0.92	↑	
Glycerol-3-phosphate	GC	2	1.27E-05	4.00E-04	1.45	↓	
Glycerophosphocholine	NMR	1	6.64E-03	9.13E-03	0.78	↓	
Glycolytic acid	GC	2	1.78E-02	4.49E-02	0.58	↓	
Histidine	LC	1	1.18E-06	1.41E-05	1.79	↑	
Hypoxanthine	NMR	1	2.49E-05	9.94E-05	1.20	↑	
Inosine monophosphate	NMR	1	2.71E-05	9.94E-05	1.36	↑	
Inosine	NMR	1	4.42E-03	6.85E-03	0.82	↑	GC
Isoleucine	GC	2	2.28E-04	2.40E-03	1.05	↑	
Kynurenine	LC	1	4.89E-04	2.20E-03	1.13	↓	
Leucine	LC	1	2.01E-02	3.45E-02	0.64	↑	GC
Malate	GC	2	7.82E-04	6.16E-03	1.14	↑	
Mannose	NMR	1	8.76E-04	1.75E-03	1.05	↑	
Methionine	LC	1	3.61E-03	8.66E-03	0.75	↑	
Myoinositol	GC	2	1.78E-02	4.49E-02	0.72	↑	
*N*-Acetylaspartic acid	LC	1	6.10E-04	2.35E-03	1.18	↑	
*N*-Acetyllysine	GC	3	6.01E-03	2.10E-02	0.80	↑	
Nicotinamide adenine dinucleotide	NMR	2	1.06E-02	1.37E-02	0.66	↑	
Nicotinamide	NMR	1	4.80E-04	1.17E-03	0.99	↑	
Phenylalanine	LC	1	4.43E-05	3.99E-04	1.20	↑	GC
Pipecolic acid	LC	1	2.46E-03	7.46E-03	0.95	↑	
Ribitol	GC	2	1.87E-02	4.54E-02	0.65	↑	
Serine	LC	1	2.86E-03	7.91E-03	0.84	↑	GC
Succinic acid	NMR	1	4.50E-04	1.17E-03	1.19	↓	GC
Tetradecanoylcarnitine (C14)	LC	1	2.76E-02	4.52E-02	0.60	↑	
Threonine	LC	1	2.49E-03	7.46E-03	0.77	↑	
Tryptophan	LC	1	2.55E-07	4.60E-06	1.87	↑	GC
Tyrosine	LC	1	3.82E-08	1.38E-06	1.72	↑	NMR
UDP-Glucose	NMR	1	7.73E-06	4.35E-05	1.43	↑	
UDP-*N*-Acetylglucosamine	NMR	1	7.91E-06	4.35E-05	1.42	↑	
Uracil	NMR	1	2.59E-03	4.38E-03	0.79	↑	
Uridine	NMR	1	4.67E-03	6.85E-03	0.93	↑	
Valine	LC	1	5.32E-03	1.20E-02	0.69	↑	GC
Xylose	GC	3	5.18E-05	8.16E-04	1.21	↑	

^a^Confidence levels of metabolite identification according to Schymanski et al. (2014); ^b^Arrows indicate an increase (↑) or decrease (↓) in KO relative to WT metabolite levels; ^c^Additional platform on which a metabolite was detected as significantly altered.

### Pathway mapping

**Fig. 3:**
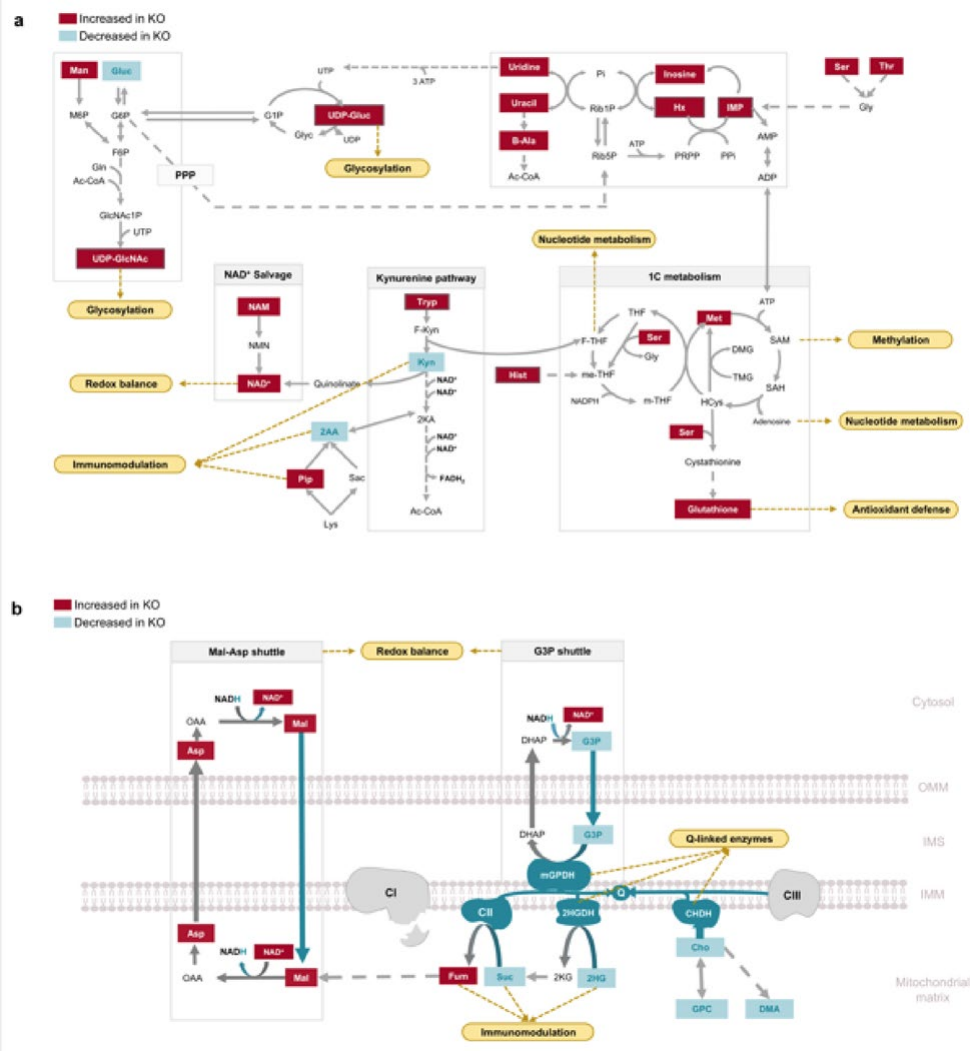
Hepatic metabolic pathway perturbations in *Ndufs4* KO mice. Significantly altered metabolites were mapped to **a** glucose metabolism i.e., the hexosamine biosynthesis and glycogen synthesis pathways, pyrimidine and purine metabolism, one-carbon metabolism, tryptophan and lysine catabolism, and NAD^+^ metabolism as well as **b** the TCA cycle, redox regulatory shuttles, and alternative Q-cycle fuelling pathways. *2AA* 2-aminoadipate, *2HG* 2-hydroxyglutarate, *2HGDH* 2-hydroxyglutarate dehydrogenase, *2KA* 2-ketoadipate, *2KG* 2-ketoglutarate, *Ac-CoA* acetylcoA, *AMP* adenosine monophosphate, *Asp* aspartate, *ATP* adenosine triphosphate, *B-Ala* betal-alanine, *CHDH* choline dehydrogenase, *Cho* choline, *DHAP* dihydroxyacetone phosphate, *DMA* dimethylamine, *F6P* fructose-6-phosphate, *F-Kyn* N-formyl-kynurenine, *F-THF* N10-formyl-tetrahydrofolic acid, *Fum* fumarate, *G1P* glucose-1-phosphate, *G3P* gycerol-3-phosphate, *G6P* glucose-6-phosphate, *GlcNac1P* N-acetyl-D-glucosamine 1-phosphate, *Gln*, glutamine;* Gluc*, glucose;* Gly*, glycine; *Glyc*, glycerol;*GPC*, glycerophosphocholine; *Hcys*, homocysteine; *His*, histidine;* Hx*, hypoxanthine; *IMP*, inosine monophosphate;*Kyn*, kynurenine;*Lys*, lysine; *M6P*, mannose-6-phosphate; *Mal*, malate; *Man*, mannose; *Met*, methionine; *me-THF*, N5N10-methylene-tetrahydrofolic acid; *mGPDH*, mitochondrial glycerol-3-phosphate dehydrogenase; *m-THF*, N5-methyl-tetrahydrofolicacid; *NAD(P)H*, nicotinamide adenine dinucleotide phosphate; *NAD+*, nicotinamide adenine dinucleotide; *NAM*, nicotinamide; *NMN*, nicotinamide mononucleotide;*OAA*, oxaloacetate; *Phe*, phenylalanine;* Pip*, pipecolate; *PRPP*, phosphoribosyl pyrophosphate; *Rib1P*, ribose-1-phosphate; *Rib5P*, ribose-5-phosphate; *Sac*, saccharopine;* SAH*, S-adenosyhomocysteine; *SAM*, S-adenosylmethionine; *Ser*, serine; *Suc*, succinate; *THF*, tetrahydrofolicacid; *Thr*, threonine; *TMG*, trimethyglycine; *Tryp*, tryptophan; *Tyr*, tyrosine;* UDP*, uridine diphosphate;* UDP-GlcNAc*, UDP-N-acetylglucosamine; *UDP-Gluc*, UDP-glucose; *UTP*uridine-5′-triphosphate

To further elucidate the biological relevance of the metabolic profiles observed in *Ndufs4* KO mice, significantly altered metabolites were mapped onto metabolic pathways. Prominent changes were identified in glucose, pyrimidine, purine, amino acid, and one-carbon metabolism (Fig. 3a) as well as the TCA cycle; redox regulatory shuttles, and alternative Q-cycle fuelling pathways (Fig. 3b).

In the *Ndufs4* KO liver, glucose levels were decreased, while mannose and two uridine diphosphate (UDP) derivatives of glucose were markedly elevated. This included UDP-*N*-acetylglucosamine, a product of the hexosamine biosynthesis pathway and UDP-glucose, involved in glycogen synthesis. The formation of these metabolites requires the pyrimidine nucleotide uridine triphosphate (UTP). Notably, the UTP precursor, uridine, along with its catabolites, uracil and β-alanine, was elevated in *Ndufs4* KO livers. Similar increases were observed for related purines, including inosine monophosphate (IMP), inosine, and hypoxanthine, which may reflect the channelling of excess uridine into to purine salvage (Balestri et al., 2007). While these shifts in glucose and nucleotide metabolism reflect genotype-dependent alterations, potential artefacts introduced during sample handling also warrant consideration. Although liver samples were rapidly snap-frozen following euthanasia, brief thawing during weighing and extraction may have introduced ex vivo metabolic changes — particularly in redox-sensitive pathways like purine degradation due to the absence of tissue perfusion (Rauckhorst et al., 2022). These artefacts could partially confound genotype-specific comparisons.

Amino acid levels were also significantly increased in *Ndufs4* KO livers, with the most pronounced elevations seen in aromatic amino acids such as tyrosine, tryptophan, histidine, and phenylalanine. Key components of one-carbon metabolism, including serine, threonine, and methionine, were also elevated, alongside glutathione, an antioxidant synthesised via the transsulfuration pathway of one-carbon metabolism. Further alterations were noted in the tryptophan and lysine catabolic pathways, where kynurenine and 2-aminoadipate levels were decreased, while pipecolate levels increased. The kynurenine pathway contributes to NAD^+^ synthesis from tryptophan. Elevations in NAD^+^ and its precursor, nicotinamide, were also evident in *Ndufs4* KO livers.

Additional metabolic changes observed in the *Ndufs4* KO liver (Fig. 3b) include decreased levels of alternative FADH_2_-linked Q-cycle substrates, such as glycerol-3-phosphate, choline, 2-hydroxyglutarate, and succinate. The latter two metabolites feed into the TCA cycle, leading to the formation of fumarate, malate, and aspartate, all of which were significantly elevated in *Ndufs4* KO livers along with *N*-acetyl aspartate. Malate, aspartate, and glycerol-3-phosphate also serve as intermediates in critical redox regulatory shuttles, which play a role in regenerating cytosolic NAD^+^.

## Discussion

This study examined the interactions between liver bioenergetics, metabolic processes, and systemic CI dysfunction in the *Ndufs4* KO mouse model at the late stage of LS. Enzyme assays confirmed that the whole-body KO of *Ndufs4* results in significant liver CI deficiency (86%), while other respiratory chain enzymes remain unaffected. These findings align with previous studies on younger *Ndufs4* KO mice, reporting a ~ 50% reduction in CI subunits (Adjobo-Hermans et al., 2020), 81% CI activity loss, and unaltered CII-IV activities in the liver (Calvaruso et al., 2012).

Our respiratory assays further revealed that this CI deficiency significantly reduces the ability of CI to contribute to combined CI + II-driven respiration, with a ~ 43% decrease detected in the CI control ratio of *Ndufs4* KO liver mitochondria. In contrast to our findings in 45 to 50-day-old *Ndufs4* KO mice, Balmaceda et al. (2024) reported a slight increase in the liver CI control ratio of younger 30 to 45-day-old *Ndufs4* KO mice. While both studies assessed respiration in isolated mitochondria, such preparations may not fully recapitulate the complexity of in vivo bioenergetic regulation, including cytosolic and systemic influences. Nevertheless, our data indicate that mouse liver intrinsically primarily relies on succinate oxidation for respiration, with ~ 85% of combined CI + II respiration resulting from CII electron input in both genotypes. Other recent studies on mouse liver have also challenged the classic view of CI as the main entry point for electrons into the respiratory chain across all tissues. Molinié et al. (2022) first reported a preferential reliance of liver mitochondria on CII-driven respiration in mice. Subsequently, similar findings were described in two CI-deficient mouse models, i.e., liver-specific *Ndufa9* KO mice (Lesner et al., 2022) and whole-body *Ndufs4* KO mice (Balmaceda et al., 2024). These studies suggested that CI is dispensable in the mouse liver under homeostatic conditions as CI deficiency had minimal impact on mitochondrial bioenergetics and metabolism.

Conversely, our metabolic data indicate that whole-body CI deficiency markedly affects liver metabolism in *Ndufs4* KO mice as the disease progresses. Elevated NAD^+^ and nicotinamide levels suggest that the liver retains its capacity to regenerate NAD^+^ despite CI deficiency, while reduced FADH_2_-linked substrates point to compensation for reduced CI electron input. With CI dysfunction, a stalled TCA cycle typically leads to increased pyruvate levels, promoting its conversion to lactate, alongside the interconversion of NADH to NAD^+^ (Esterhuizen et al., 2017). However, lactic acid was not significantly altered in KO livers, compared to WTs (*p* = 0.791). The reduced CI control ratio in liver mitochondria further indicates significantly impaired CI respiration, thus raising the question of how the liver retains its capacity to regenerate NAD^+^ and lower reductive stress. One possible explanation is that NADH may be diverted to ketogenesis, facilitating the formation of 3-hydroxybutyric acid, which is then exported to serve as an energy source for other organs (Mooli & Ramakrishnan, 2022). Alternatively, the lack of elevated lactic acid in KO livers may suggest that lactate is used as a gluconeogenic precursor, supporting glucose production while regenerating cytosolic NAD^+^. Further research, including metabolic flux analysis, is needed to explore these hypotheses.

However, it is important to note that these metabolic changes occur in the context of a 12-hour fast, which introduces physiological shifts that warrant careful consideration. While fasting is a standard approach in rodent studies to reduce variability from feeding and postprandial fluctuations (Jensen et al., 2013), it triggers pronounced hepatic metabolic changes driven by elevated glucagon and reduced insulin signalling (Carper et al., 2020). A 12-hour fast in mice results in hepatic glycogen depletion, leading to increased gluconeogenesis and ketogenesis, both fuelled by enhanced fatty acid oxidation (Carper et al., 2020; Fu et al., 2024; Sokolović et al., 2008). These changes are accompanied by shifts in redox balance that may elevate NAD^+^ levels (Rodgers et al., 2005) while enhanced proteolysis and amino acid catabolism occurs to supply substrates for glucose production and the TCA cycle (Carper et al., 2020; Fu et al., 2024; Sokolović et al., 2008).

These fasting-induced adaptations complicate the interpretation of our findings by potentially confounding genotype-specific effects. Fasting may unmask mitochondrial deficiencies that are less apparent in the fed state, or conversely, partially compensate for CI dysfunction via enhanced fatty acid oxidation and ketogenesis. Such adaptations could shift the metabolic profile toward a stress-adapted rather than basal state, potentially explaining the preserved NAD⁺ regeneration despite impaired CI activity. Nonetheless, comparing *Ndufs4* KO and WT mice under identical fasting conditions remains a valid approach for identifying genotype-dependent metabolic responses to nutrient stress and for revealing pathways most sensitive to mitochondrial dysfunction under energetic challenge. Further studies comparing fed and fasted *Ndufs4* KO mice could help determine whether the observed metabolic changes are intrinsic to CI deficiency or amplified by fasting. Such comparisons would aid in distinguishing basal metabolic phenotypes from secondary adaptations to nutrient deprivation, thereby clarifying how nutritional status influences disease progression and possibly therapeutic responsiveness.

Furthermore, the accumulation of most amino acids, particularly those linked to one-carbon metabolism, suggests increased metabolic demands in the *Ndufs4* KO liver. Our earlier analysis of urine from the same animals used in this study (Terburgh et al., 2021), showed reduced excretion of these amino acids, indicating that their hepatic accumulation likely reflects cellular stress rather than a metabolic blockade. This accumulation may also have systemic implications via inter-tissue mechanisms. As described by Uno et al. (2015), elevated liver amino acids activate mTORC1/S6K signalling, modulating lipid metabolism through a neuronal pathway that leads to hypertriglyceridemia via the downregulation of adipose lipoprotein lipase. This mechanism aligns with the elevated serum triglycerides reported in liver-specific *Ndufs4*-KO mice (Jin et al., 2014) and the benefits of disrupting liver-specific S6K1 in whole-body *Ndufs4* KO mice (Ito et al., 2017).

Moreover, the significant elevation we observed in *Ndufs4* KO liver uridine, UDP-glucose, and UDP-*N*-acetylglucosamine (UDP-GlcNAc) indicates a shift in glucose metabolism toward anabolic pathways, including glycogen synthesis and the hexosamine biosynthesis pathway, which produce precursors for protein glycosylation (Führing et al., 2015; Zhang et al., 2014). The availability of UDP-GlcNAc regulates *O*-GlcNAcylation, a glycosylation process that functions as a nutrient sensor, integrating metabolism, signalling, and transcription to regulate cellular functions, including glucose homeostasis in the liver (Hu et al., 2024; Zhang et al., 2014). While elevated O-GlcNAcylation is an adaptive response to various stressors, its persistent dysregulation has been implicated in mitochondrial dysfunction, neurodegeneration, and inflammatory diseases. Cross regulation between *O*-GlcNAcylation and mTOR signalling has also been observed in metabolic disorders (Very et al., 2018).

Thus, the metabolic changes in *Ndufs4* KO mouse liver highlight a complex interplay between stress adaptation and potential metabolic dysfunction with systemic implications that may contribute to disease progression.

## Conclusions

To conclude, we have shown that despite a significant CI deficiency, overall liver respiration remains largely unaffected in the *Ndufs4* KO mouse as this tissue mostly relies on CII-driven respiration. However, whole-body CI deficiency does markedly affect liver metabolism as LS progresses. Using hypothesis- generating metabolic profiling, we identified key areas of liver metabolism to further investigate in the context of LS. Given the liver’s pivotal role in immunomodulation, interorgan metabolism, and the liver-brain axis, it is imperative to unravel the specific mechanisms underlying the observed metabolic alterations. While this study did not directly assess O-GlcNAcylation or mTOR signalling, it is suggestive of alterations in these pathways. Future work investigating their roles and potential crosstalk in LS could offer valuable mechanistic insights that could not only shed light on disease progression but also open avenues for targeted therapeutic interventions.

## Data Availability

The data from this study are available through the NIH Common Fund’s Data Repository and Coordinating Center, supported by NIH grant U01-DK097430, at the Metabolomics Workbench website (http://www.metabolomicsworkbench.org) with study ID ST003766.
